# Measuring patient centeredness with German language Patient-Reported Experience Measures (PREM)–A systematic review and qualitative analysis according to COSMIN

**DOI:** 10.1371/journal.pone.0264045

**Published:** 2022-11-29

**Authors:** Andre L. Mihaljevic, Colette Doerr-Harim, Eva Kalkum, Guido Strunk

**Affiliations:** 1 Department of General and Visceral Medicine, University Hospital Ulm, Ulm, Germany; 2 Clinical Trial Centre, Department of Surgery (*ulmCARES*), University Hospital Ulm, Ulm, Deutschland; 3 Study Centre of the German Society of Surgery, Heidelberg, Germany; 4 Complexity-Research, Wien, Austria; Murcia University, Spain, SPAIN

## Abstract

**Background:**

Patient centeredness is an integral part of the quality of care. Patient-reported experience measures (PREMs) are assumed to be an appropriate tool to assess patient-centredness. An evaluation of German-speaking PREMs is lacking.

**Objective:**

To perform a systematic review and qualitative analysis of psychometric measurement qualities of German-language PREMs using for the first time a comprehensive framework of patient centredness.

**Methods:**

A systematic literature search was performed in Medline, PsycInfo, CINHAL, Embase, Cochrane database (last search 9^th^ November 2021) for studies describing generic, surgery- or cancer care-specific PREMs. All questionnaires that were developed in or translated into German were included. The content of the included PREMs was evaluated using a comprehensive framework of patient centredness covering 16 domains. Baseline data of all PREM studies were extracted by two independent reviewers. Psychometric measurement qualities of the PREMs were assessed using current COSMIN guidelines.

**Results:**

After removal of duplicates 3,457 abstracts were screened, of which 3,345 were excluded. The remaining 112 articles contained 51 PREMs, of which 12 were either developed in (4 PREMs) or translated into German (8 PREMs). Eight PREMs were generic (NORPEQ, PPE-15, PEACS, HCAHPS, QPPS, DUQUE, PEQ-G, Schoenfelder et al.), 4 cancer care-specific (EORTC IN-PATSAT32, PSCC-G, Danish National Cancer Questionnaire, SCCC) and none was surgery-specific. None of the PREMs covered all domains of patient-centeredness. Overall rating of structural validity was adequate only for PEACS and HCAHPS. High ratings for internal consistency were given for NORPEQ, Schoenfelder et al., PSCC-G and the SCCC. Cross-cultural validity for translated questionnaires was adequate only for the PSCC-G, while reliability was adequately assessed only for the EORTC IN-PATSAT32. Due to a lack of measurement gold standard and minimal important change, criterion validity and measurement invariance could not be assessed for any of the PREMs.

**Conclusion:**

This is the first systematic review using a comprehensive framework of patient centredness and shows that none of the included PREMs, even those translated from other languages into German, cover all aspects of patient centredness. Furthermore, all included PREMS show deficits in the results or evaluation of psychometric measurement properties. Nonetheless, based on the results, the EORTC IN-PATSAT32 and PSCC-G can be recommended for use in cancer patients in the German-language region, while the German versions of the HCAHPS, NORPEQ, PPE-15 and PEACS can be recommended as generic PREMs.

**Trial Registration:**

**Registration.** PROSPERO CRD42021276827.

## Introduction

Improving the patient centredness (PC) of healthcare has been a main objective of healthcare politics over the last decades, including German-speaking countries [1, 2]. PC has been defined as one of six domains of the quality of care by the Institute of Medicine (IOM), next to safety, effectiveness, timeliness, efficiency and equitability [3] (S1 Fig). However, patients frequently experience a lack of PC in many fields of healthcare [4].

Furthermore, the dimensions of PC have not been clearly defined and several models have been proposed in the past (S1 Table). A systematic review has identified 15 dimensions of PC [5]: patient as a unique person, biopsychosocial perspective, essential characteristics of the clinician, patient involvement in care, involvement of family and friends, physical support, emotional support, clinician-patient communication, patient empowerment, patient Information, access to care, integration of medical and non-medical care, coordination and continuity of care, teamwork and teambuilding, clinician-patient relationship. The influential Picker model contains an additional dimension termed “effective treatment by trustworthy and qualified personnel” [6] (S1 Table).

Several methods have been proposed to measure PC in clinical practice [7]. Assessment via questionnaires termed *Patient-Reported Experience Measures* (PREM) is most frequent as they permit a standardized appraisal of PC. PREMs aim to measure PC via the experience of patients in a certain healthcare context. Depending on this healthcare context four different categories of PREMs can be distinguished, although they partially overlap:

- *generic PREMs* measure general aspects of PC and can be applied across multiple healthcare settings and disciplines.
- *discipline-specific PREMs* assess the PC within a certain discipline. For example, surgery-specific PREMs measure, among others, aspects of PC specific to surgical disciplines e.g. pain.
- *healthcare pathway-specific PREMs* measure the PC across a specific healthcare pathway. For example, a *cancer care -specific PREM* measures aspects of PC important to cancer patients irrespective of treatment (surgery, chemotherapy, radiotherapy) and healthcare setting (in-hospital or as outpatient).
- *disease-specific PREMs* aim to measure the PC of healthcare for patients with a specific disease, e.g., breast cancer.

According to Bull et al. [8] PREMs (a) enable patients to reflect on their care experience; (b) provide patient-level information to drive service quality improvement; (c) serve as quality indicators for public reporting and benchmarking. PREMs need to be distinguished from *Patient-Reported Outcome Measures* (PROM). PROMs measure medical outcomes, rather than dimensions of PC and therefore, address different domains of healthcare in the IOM quality of care model (S2 File). Like PROMs, however, PREMs need to have adequate psychometric measurement properties (e.g., validity, reliability) to accurately assess PC. However, non-standardized PREMs or PROMs are frequent [9] and constitute a waste of resources [10]. In order to avoid such research waste, the COSMIN (COnsensus-based Standards for the selection of health Measurement INstruments) group has defined internationally accepted guidelines for assessing psychometric properties of patient-reported measures [11]. These guidelines are applicable to PROMs as well as PREMs and may help to identify reliable and valid measurement tools to assess PC.

Contrary to other languages no comprehensive review is available for German PREMs. Furthermore, the psychometric properties of German PREMs have not been evaluated yet. Therefore, the aim of the study is to perform a systematic review and qualitative analysis of generic, surgery-specific and cancer care-specific German PREMs using the internationally accepted COSMIN guidelines [11]. In addition, the current study uses for the first time the comprehensive model of patient centredness proposed by Scholl et al. to evaluate the content of included PREMs [5].

## Material and methods

This systematic review is reported according to current PRISMA guidelines [12]. A PRISMA checklist is attached (S2 Table). The review has been registered (PROSPERO CRD42021276827). No funding has been received for this work. A review protocol had been written prior to data extraction.

### Eligibility criteria

Studies that described or analysed the development, validation, or measurement of generic, surgery-specific or cancer healthcare pathway-specific PREMs were included in this systematic review. Included studies needed to be published in a peer-reviewed journal. During the initial search no language limitations were set. The following exclusion criteria were set:

1. Studies that described general satisfaction questionnaires that did not cover aspects of patient experience.
2. Studies measuring patient expectation rather than patient experience
3. Studies that measured single aspects of patient-centeredness and were thus no multidimensional PREMs
4. Studies describing questionnaires for physicians or proxies and not for patients themselves (i.e., were not patient-reported)
5. Studies containing PREMs that were not generic, surgery-specific or cancer healthcare pathway-specific. Therefore, disease-specific PREMs were excluded.

### Information sources

The following information sources were searched:

- EBM Reviews—Cochrane Database of Systematic Reviews 2005 to November 9, 2021, EBM Reviews—Health Technology Assessment 4th Quarter 2016
- Embase 1980 to 2021 Week 40
- Ovid MEDLINE(R) ALL 1946 to November 9, 2021
- APA PsycInfo 1806 to November Week 2 2021
- CINHAL November 9, 2021

The last search was performed on 9^th^ November 2021. An existing literature review on PREMs by Bull et al. was used as template [8]. This publication searched PREMs until 31^st^ March 2018. Therefore, our search was limited to 2018 till November 2021. The results by Bull et al. were included in this systematic review.

### Search

The search algorithm is described in detail in supplement 4 (S1 File). It is an adaptation of the search algorithm described by Bull et al. [8]. Additional studies were identified by reference searching and full text reading.

### Study selection

The references of all generic, surgery-specific or cancer healthcare pathway-specific PREMs were imported into the citation program Zotero (www.zotero.org; Version 5.0.96.2). Duplicates were identified and merged either with the find duplicates function in Zotero or by hand. Titles and abstracts of all articles were read by two reviewers (AMi, CDH) and those studies not fulfilling eligibility criteria were removed. In a next step, the fulltext articles of all remaining studies were read, to decide which articles fulfil eligibility criteria. Fulltext as well as references were screened to identify additional PREMs. For all non-German PREMs fulfilling the eligibility criteria additional searches were performed in the above-mentioned databases to identify German translations. In addition, Google and google scholar were search with the name of the PREM in combination with “German translation” or “cross-cultural validation” to identify German translations. Only PREMs developed in German or for which a German language translation existed were considered for further analyses.

### Data collection process

Data was extracted on prespecified forms by two reviewers (AMi, CDH). The following data items were collected: author, year, journal, PREM acronym, country of origin, description of the PREM, method and timepoint of PREM collection, number of questions, PREM domains according to original article, presence of a German version, free text commentary.

### Assessment of psychometric properties and data synthesis

Psychometric properties were used according to the COnsensus-based Standards for the selection of health Measurement INstruments (COSMIN) terminology [13]. Qualitative analysis of psychometric properties was done in according to current COSMIN guidelines [14, 15] (www.cosmin.nl). The evaluation of content validity was done according to Terwee et al. [11]. We used the 16 PC dimensions described in S1 File to assess content validity. The following psychometric properties were analysed: (1) content validity; (2) structural validity; (3) internal consistency; (4) measurement invariance / cross-cultural validity; (5) reliability; (6) measurement error; (7) criterion validity; (8) hypothesis testing for construct validity. Table 1 shows the COMSIN assessment criteria used in this study. For overall evaluation “+” marks adequate, “-”inadequate and “?” unclear psychometric properties. A detailed description of the methods can be found in S2 File.

**Table 1:** Criteria for overall assessment of psychometric properties according to COSMIN (Mokkink et al. 2018, COSMIN User Manuel).

Measurement property	Rating	Criteria
Structural validity	+	**CTT:** CFA: CFI or TLI or comparable method >0.95 OR RMSEA <0.06 OR SRMR <0.082 **IRT/Rasch:** No violation of unidimensionality3: CFI or TLI or comparable measure >0.95 OR RMSEA <0.06 OR SRMR <0.08 AND no violation of local independence: residual correlations among the items after controlling for the dominant factor <0.20 OR Q3’s < 0.37 AND no violation of monotonicity: adequate looking graphs OR item scalability >0.30 AND adequate model fit: IRT: χ2 >0.01 Rasch: infit and outfit mean squares ≥ 0.5 and ≤ 1.5 OR Z standardized values > ‐2 and <2
	**?**	CTT: Not all information for ‘+’ reported IRT/Rasch: Model fit not reported
	**-**	Criteria for ‘+’ not met
**Internal consistency**	**+**	At least low evidence for sufficient structural validity AND Cronbach’s alpha(s) ≥ 0.70 for each unidimensional scale or subscale
	**?**	Criteria for “At least low evidence for sufficient structural validity” not met
	**-**	At least low evidence for sufficient structural validity5 AND Cronbach’s alpha(s) < 0.70 for each unidimensional scale or subscale
**Measurement invariance (cross-cultural validity)**	**+**	No important differences found between group factors (such as age, gender, language) in multiple group factor analysis OR no important DIF for group factors (McFadden’s R2 < 0.02)
	**?**	No multiple group factor analysis OR DIF analysis performed
	**-**	Important differences between group factors OR DIF was found
**Reliability**	**+**	ICC or weighted Kappa ≥ 0.70
	**?**	ICC or weighted Kappa not reported
	**-**	ICC or weighted Kappa < 0.70
**Measurement error**	**+**	SDC or LoA < MIC
	**?**	MIC not defined
	**-**	SDC or LoA > MIC
**Criterion validity**	**+**	Correlation with gold standard ≥ 0.70 OR AUC ≥ 0.70
	**?**	Not all information for ‘+’ reported
	**-**	Correlation with gold standard < 0.70 OR AUC < 0.70
**Hypothesis testing for construct validity**	**+**	The result is in accordance with the hypothesis
	**?**	No hypothesis defined (by the review team)
	**-**	The result is not in accordance with the hypothesis

“+” = sufficient,”–”= insufficient, “?” = indeterminate.

AUC: Area under the curve. CTT: classical test theory. CFA: confirmatory factor analysis, CFI: comparative fit index. DIF: differential item functioning. ICC: interclass correlation coefficient. IRT: item response theory. MIC: minimal important change. LoA: limits of agreement. RMSEA: Root Mean Square Error of Approximation. SDC: smallest detectable change. SRMR: Square Root Mean Residuals. TLI: Tucker‐Lewis index

## Results

### Study selection and study characteristics

**Fig 1:**
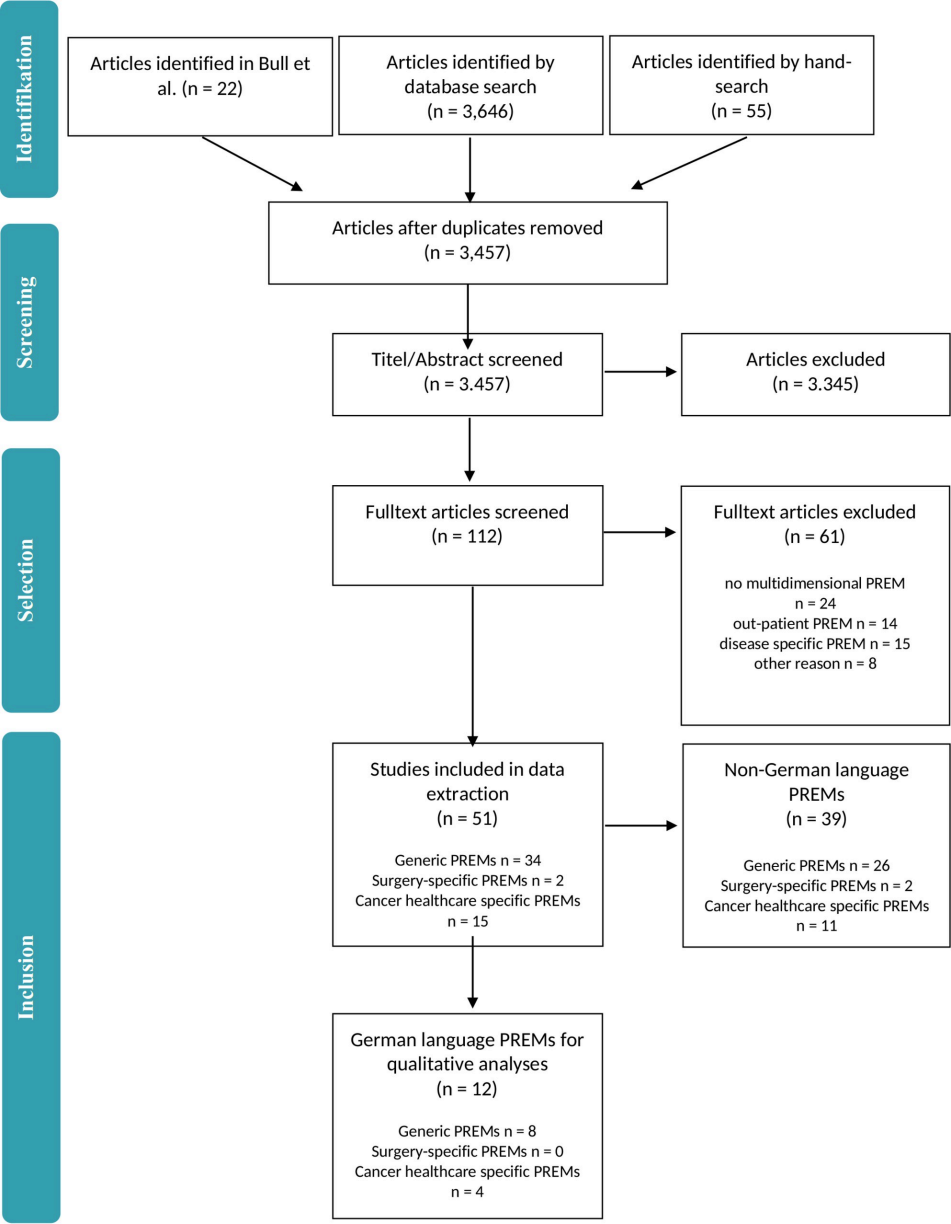
PRISMA Flow-chart of included studies.

By database searching 3,646 articles were identified. An additional 55 potential studies were found by hand-search and 22 PREMs were added through the systematic review by Bull et al. [8] (Fig 1). After removal of duplicates 3,457 articles remained for screening. After screening abstracts and titles, 112 fulltext articles remained of which 51 articles fulfilled all eligibility criteria. Excluded full text articles are listed in S4 Table. Twelve of these 51 articles described German PREMs [16–27] (Table 2). Details of the non-German PREMs are listed in S3 Table. Of the 12 German-language PREMs, eight were generic [16, 18–20, 24–27] and four were cancer-care specific PREMs [17, 21–23]. No surgery-specific German-language PREM was identified. One of the German-language PREMs *(Quality from the Patients‘ Perspective*, *QPP)* is available only in its short-form *(short-form*, *QPPS)* [28]. The PREMs are either translations into German [16–22] or were developed in German [23–26]. Study characteristics can be found in Table 2.

**Table 2:** Study characteristics of German-language Patient-Reported Experience Measures (PREMs).

	Author	Ref.	Year	Acronym	Country	Description	Method (timepoint) of assessment	Number of items	Domains	Comments
**Generic PREMs**										
**1**	Oltedal S, Garratt A, Bjertnaes Ø, Bjørnsdottìr M, Freil M, Sachs M.	Scandinavian Journal of Public Health, 2007; 35: 540–547	2007	NORPEQ	Norway	Further publications ins Skudal KE, Garratt AM, Eriksson B, Leinonen T, Simonsen J, Bjertnaes OA, BMJ Open. 2012;2(3):11.	paper-based (within 7 weeks after discharge)	8	1. Whether doctors were understandable 2. Doctors’ professional skills 3. Nurses’ professional skills 4. Nursing care 5. Whether doctors and nurses were interested in the patients’ problems 6. Information on tests	German version used in Groene O, et al. Patient Experience Shows Little Relationship with Hospital Quality Management Strategies. PLoS One. 2015 Jul 7;10(7.
**2**	Jenkinson C, Coulter A, Bruster S.	Int J Qual Health Care. 2002;14(5):6.	2002	Picker PPE-15	Great Britain	“(1) to determine what factors are most likely to influence satisfaction with care and willingness to recommend hospital services to others and (2) to explore the extent to which satisfaction is a meaningful indicator of patients’ experience of healthcare services.”	paper-based (within 1 month after discharge)	15	1. information and education 2. coordination of care 3. physical comfort 4. emotional support 5. respect for patient preferences 6. involvement of family/friends 7. continuity of care.	
**3**	Noest S, Ludt S, Klingenberg A, et al.	Int J Qual Health Care. 2014;26(3):10.	2014	PEACS	Germany	“To develop and validate a generic questionnaire to evaluate experiences and reported outcomes in patients who receive treatment across a range of healthcare sectors”	paper-based (after discharge; exact timepoint not specified)	59 (total) 40 (short-form)	1. Preliminary care 2. Shared decision-making at indication 3. Patient education and information 4. Nursing staff 5. Accessible physicians 6. Pain therapy 7. Institutional treatment and transition 8. Information at discharge follow-up Outcome	
**4**	Hays RD, Shaul JA, Williams VS, et al.	Med Care. 1999;37(3 Suppl):MS22‐31.	1999	HCAHPS CAHPS	USA	Further references in: 1.) Keller S, O’Malley A, Hays RD, Mathew RA, Zaslavsky AM, Hepner AM, et al. Methods used to streamline the CAHPS Hospital Survey. Health Serv Res. 2005;40:2057–77. 2.) Hargraves JL, Hays RD, Cleary PD. Psychometric properties of the Consumer Assessment of Health Plans Study (CAHPS) 2.0 adult core survey. Health Serv Res. 2003;38(6 Pt 1):1509‐1527. 3.) Hays RD, Martino S, Brown JA, et al. Evaluation of a care Med Care Res Rev. 2014;71(2):192‐202 4.) Sofaer S, Crofton C, Goldstein E, Hoy E, Crabb J. Health Serv Res. 2005;40:2018–36 5.) Agency for Healthcare Research and Quality. HCAHPS Fact Sheet CAHPS Hospital Survey. USA; 2012	paper-based or telephone-interview	29	4 Global rating items 1. Health plan 2. Quality of care 3. Personal doctor 4. Specialist 10 domains of health plan performance 1. Getting care you need 2. Getting care without long wait 3. Communication of doctors 4. Doctors spending enough time with their patients 5. Prevention 6. Office staff 7. Customer service 8. Reasonable paperwork 9. Finding a personal doctor 10. Referrals to specialists	German translation in Squires A, Bruyneel L, et al. Cross-cultural evaluation of the relevance of the HCAHPS survey in five European countries. Int J Qual Health Care. 2012 Oct;24(5):470–5. Further references in: 6.) Giordano L, Ellito M, Goldstein E, Lehrman E, Spencer P. Med Care Res Rev. 2009;67:27–37 7.) Levine RE, Fowler FJ, Brown JA. Health Serv Res. 2005;40:2037–56 8.) O’Malley AJ, Zaslavsky AM, Hays RD, Hepner KA, Keller S, Cleary PD. Health Serv Res. 2005;40:2078–94
**5**	Larsson BW, Larsson G.	J Clin Nurs. 2002;11:681–7.		QPP-S (Quality from the Patients’ Perspective Shortened)	Sweden	short-form of the QPP	paper-based (at discharge)	24	1. Medical-technical competence 2. Physical technical conditions 3. Identity-orientated approach 4. Socio-cultural atmosphere	German translation in 1.) Singer S, Götze H, Möbius C, Witzigmann H, Kortmann RD, Lehmann A, Höckel M, Schwarz R, Hauss J. Langenbecks Arch Surg. 2009 Jul;394(4):723–31. doi: 10.1007/s00423-009-0489-5. 2) Singer S, Danker H, Meixensberger J, Briest S, Dietz A, Kortmann RD, Stolzenburg JU, Kersting A, Roick J. J Cancer Res Clin Oncol. 2019 Nov;145(11):2845–2854.
**6**	Rosa Suñol, Oliver Groene, the DUQuE Project Consortium	http://www.duque.eu/index.php?page = participating-countries		DUQUE (Deepening our Understanding of Quality Improvement in Europe)	Czech Republic, France, Germany, Poland, Portugal, Spain, Turkey, Great Britain	Set of patient-reported experience measures including • a generic 6-item measure of patient experience (NORPEQ) • a 3-item measure of patient-perceived discharge preparation (Health Care Transition Measure) • perceived involvement in care (from Commonwealth Fund sicker patients survey) • hospital recommendation (from HCAHPS)	paper-based	24	1. Generic patient experience 2. Perceived patient involvement 3. Hospital recommendation Perceived continuity of care	The German version of the questionnaire has not yet been validated. Further references in: Groene O, Arah OA, Klazinga NS, Wagner C, Bartels PD, Kristensen S, Saillour F, Thompson A, Thompson CA, Pfaff H, DerSarkissian M, Sunol R. Patient Experience Shows Little Relationship with Hospital Quality Management Strategies. PLoS One. 2015 Jul 7;10(7):e0131805.
**7**	Christoph Gehrlach, Thomas Altenhöner, David Schwappach	Der Patients’ Experience Questionnaire. Patientenerfahrungen vergleichbar machen. Verlag Bertelsmann Stiftung. ISBN: 978-3-86793-021-5. 2009.	2009	PEQ (German) (Patient Experience Questionnaire)	Switzer-land, Germany	Developed in Switzerland and Germany	paper-based (14 days till 8 weeks after discharge. Two reminders after 14 and 28 days)	15	1. Care by physicians (*Ärztliche Versorgung*) 2. Care by nurses (*Pflegerische Betreuung*) 3. Organisation and Service 4. Recommendation (*Weiterempfehlung*)	There is a birth-specfic version (PEQ-Geburt)
**8**	Tonio Schoenfelder, Joerg Klewer, Joachim Kugler	International Journal for Quality in Health Care, Volume 23, Issue 5, October 2011, Pages 503–509,	2011	N/A	Germany	“Rates elements of satisfaction for cancer patients.”	paper-based (after treatment)	28	1. Global patient satisfaction 2. Medical aspects of care 3. Performance of service Patient expectations	Application in surgery: Schoenfelder T, Klewer J, Kugler J. Factors associated with patient satisfaction in surgery: the role of patients’ perceptions of received care, visit characteristics, and demographic variables. J Surg Res. 2010 Nov;164(1):e53-9.
**cancer care-specific PREMs**										
**1**	Brédart A, Ravazi D, Delvaux N, Goodman V, Farvacques C, Van Heer C.	A comprehensive assessment of satisfaction with care for cancer patients. Support Care Cancer. 1998;6(6):518–23	1998	EORTC IN-PATSAT32 (in-patient satisfaction with cancer care)	Europe	For in-hospital cancer patients independent of treatment discipline	paper-based (2 weeks after discharge)	32	1. Technical quality 2. Interpersonal manner 3. Communication skills 4. Coordination 5. Waiting time 6. Continuity 7. Availability 8. Access 9. Physical environment	To assess the *patient experience* of cancer patients treated in hospital.
**2**	Bokemeyer F, Lange-Drenth L, Jean-Pierre P, Schulz H, Bleich C.	Psychometric evaluation of the German version of the Patient Satisfaction with Cancer-related Care questionnaire. BMC Health Serv Res. 2020 Oct 27;20(1):983.	2020	PSCC-G (German Version of the Patient Satisfaction with Cancer Related Care)	Germany	Translation of the PSCC with additional questions from the PASCQOC questionnaire and the RESPERES-G questionnaire	paper-based	18	1. Patient as a unique person 2. Essential characteristics of the clinician 3. Patient involvement in care 4. Emotional support 5. Clinician-patient communication 6. Family and friends 7. Patient empowerment 8. Patient information 9. Access to care 10. Integration of medical and non-medical care 11. Coordination of care 12. Teamwork 13. Clinician-patient relationship 14. Effective treatment	- German translation- Original PSCC: Jean-Pierre P, Fiscella K, et al. Cancer. 2011;117(4):854–61. and Jean-Pierre P, Fiscella K, et al., Support Care Cancer. 2012;20(9):1949–56. - Additional questions from Recherché Evaluative sur la Performance de Réseau de Santé-German (RESPERES-G) and Patient Satisfaction and Quality in Oncological Care (PASQOC)
**3**	Rudolph C, Petersen GS, Pritzkuleit R, Storm H, Katalinic A.	The acceptance and applicability of a patient-reported experience measurement tool in oncological care: a descriptive feasibility study in northern Germany. BMC Health Serv Res. 2019 Nov 1;19(1):786.	2019	Danish National Cancer Patient Questionnaire	Denmark	Translation of the Danish National Cancer Patient Questionnaire (Version 2017) and Feasibility testing in colorectal and breast cancer patients	paper-based (6 till 9 months after diagnosis)	99	1. Diagnostic delay and satisfaction with diagnostic phase 2. Information & involvement 3. Continuity of care 4. Help & support 5. Discharge and overall treatment	German translation. „…modifications of questions heavily related to the Danish health care system, especially referring to the diagnostic phase, are necessary “
**4**	Esser P, Sautier L, Sarkar S, Schilling G, Bokemeyer C, Koch U, Friedrich M, Defossez G, Mehnert-Theuerkauf A.	Development and preliminary psychometric investigation of the German Satisfaction with Comprehensive Cancer Care (SCCC) Questionnaire. Health Qual Life Outcomes. May 17;19(1):147.	2021	SCCC Satisfaction with Comprehensive Cancer Care	Germany	Assessment of patient satisfaction with „*Comprehensive Cancer Care*“	paper-based (in-hospital and outpatients. Timepoint of assessment not defined)	32	1. Competence and human quality of physician (Competence) 2. Quality and quantity of information (Information) 3. If needed: access to psychosocial support services (Access) 4. Psychological support by medical staff (Support) 5. Global satisfaction with medical care 6. Global satisfaction with psychosocial care	Based on the French REPERES-60 questionnaire (for breast cancer patients). Additional questions evaluating psychosocial support.

### Content validity

Content validity was evaluated using he 16 dimensions of PC outlined in the introduction (Table 3). None of the questionnaires covers all aspects of PC. An overview of the methodological quality of studies analyzing the content validity according to COSMIN can be found in Table 4. Alle 12 included PREMs exhibit sufficient relevance. Because of the lack of content dimensions all questionnaires have deficits in *comprehensiveness* (Table 4). Understandability of questionnaires is adequate in most cases. However, the methodological quality of content validity studies varies widely from low (Schoenfelder, DUQUE) to high (Picker, HCAHPS, PEQ, EORTC IN-PATSAT32, PSCC-G) (Table 4).

**Table 3:** Overview of content domains of German-language PREMs.

	PREM Domain	HCAHPS	PEACS	PPE-15	NORPEQ	QPP*	QPPS	DUQUE	PEQ (German)	Schoen- felder et al.	EORTC IN-PATSAT32	PSCC-G	Danish National CPQ	SCCC
**1**	patient as a unique person	-	-	-	-	+	+	-	+	-	+ / -	+	+	+
**2**	biopsychosocial perspective	-	-	-	-	+ / -	-	-	-	+	-	-	-	+
**3**	essential characteristics of the clinician	-	-	-	+ / -	-	-	+ / -	-	+	+	+	+	+
**4**	patient involvement in care	-	+	+	+	+	+ / -	+	+	+	-	+ / -^4^	+	+
**5**	involvement of family and friends	-	+	+	-	+		+	-	-	-	+ / -^4^	-	-
**6**	physical support	+	+	+	+/-	+	+	-	-	-	+		+	+
**7**	emotional support	-	+	+	+/-	+ / -	+ / -	+	-	-	+	+ / -	+	+
**8**	clinician-patient communication	+	+	+	+	+ / -	+ / -	+	-	+	+	+	+	+
**9**	patient empowerment	-	+	-	-	+ / -	-	+	-	-	-	+	-	+
**10**	patient Information	+	+	-	+	+	+ / -	+	+	+	+	+ / -^4^	+	+
**11**	access to care	-	+	-	-	-	-	-	+	-	+ / -	+	-	+
**12**	integration of medical and non-medical care	-	+	-	-	-	-	-	-	-	-	+	-	+ / -
**13**	coordination and continuity of care	+/-	+	+	-	-	-	+ / -	+ / -	+	-	+	+	+ / -
**14**	teamwork and teambuilding	-	+	-	-	-	-	-	-	-	+ /—(only nursing care)	+^1^	-	-
**15**	clinician-patient relationship	+/-	+	-	+/-	+	+ / -	+ / -	+	-	+	+	+	+
**16**	effective treatment	-	+	-	+ / -	+	+	-	+	+	+	+	-	+ / -

**Table 4:** Rating of content validity of German PREMs according to COSMIN.

		Relevance					Comprehensiveness	Comprehensibility						
		1	2	3	4	5	6	7	8	9	10			
		Are the included items relevant for the construct of interest?	Are the included items relevant for the target population of interest?	Are the included items relevant for the context of use of interest?	Are the response options appropriate?	Is the recall period appropriate?	Are no key concepts missing?	Are the PROM instructions understood by the population of interest as intended?	Are the PROM items and response options understood by the population of interest as intended?	Are the PROM items appropriately worded?	Do the response options match the question?	Quality of Evidence*	Quality of methods*	Comment
generic PREMs		** **	** **	** **	** **	** **	** **	** **	** **	** **	** **	** **	** **	** **
**1**	**NORPEQ**	**+**	**+**	**+**	**+**	**+**	**-**	**+**	**+**	**+**	**+**	moderate	moderate	no cross-cultural validation in German
**2**	**Picker PPE-15**	**+**	**+**	**+**	**+**	**+**	**+/-**	**+**	**+**	**+**	**+**	moderate	high	unclear whether surgical patients were involved in design of the questionnaire
**3**	**PEACS**	**+**	**+**	**+**	**+**	**?**	**+**	**+**	**+**	**+**	**+**	high	moderate	
**4**	**HCAHPS**	**+**	**+**	**+**	**+**	**+/-**	**-**	**+**	**+**	**+**	**+**	moderate	high	
**5**	**QPPS**	**+**	**+**	**+**	**+**	**+**	**-**	**?** ***	**?** ***	**?** ***	**+**	high**	high**	Short-version of the QPP. No surgical patients involved in the design of the questionnaire. No cross-cultural validation in German.
**6**	**DUQUE**	**+**	**+**	**+**	**+**	**?**	**-**	**?** ***	**?** ***	**?** ***	**+**	low	low	
**7**	**PEQ (German)**	**+**	**+**	**+**	**+**	**+**	**-**	**+**	**+**	**+**	**+**	high	high	
**8**	**Schoenfelder et al.**	**+**	**+**	**+**	**+**	**?**	**-**	**+**	**+**	**+**	**+**	low	low	
Cancer-care specific PREM** **														
**1**	**EORTC IN-PATSAT32**	**+**	**+**	**+**	**+**	**+**	**+ / -**	**+**	**+**	**+**	**+**	high	high	
**2**	**PSCC**	**+**	**+**	**+**	**+**	**+**	**+**	**+**	**+ / -**	**+**	**+**	moderate	high	
**3**	**Danish National Cancer Patient Questionnaire**	**+**	**+**	**+ / -** ****	**+**	**+ / -**	**+ / -**	**+ / -**	**+**	**+**	**+**	low*** (German translation)	low*** (German translation)	
**4**	**PASQOC**	**+**	**+**	**+**	**+**	**?**	**+ / -** *****	**+**	**+**	**+ / -**	**+**	moderate	moderate	
**5**	**SCCC**	**+**	**+**	**+**	**+**	**?**	**+ / -**	**+/-**	**+**	**+/-**	**+**	moderate	moderate	Based on the REPERES‐60, which was designed for breast cancer patients.

* high, moderate, low, very low

** rating is based on the long-version (QPP) rather than QPPS

*** lack of validation study in German

**** authors recommend to omit several items in the German version

*****designed for the outpatient setting

### Structural validity

“Structural validity refers to the degree to which the scores of a PROM (or PREM) are an adequate reflection of the dimensionality of the construct to be measured” [11]. A summary table of the findings on structural validity can be found in Table 5. Not all necessary data on confirmatory factor analyses according to current COSMIN guidelines were available to rate the NORPEQ or the questionnaire by Schoenfelder et al. (Table 5). The PPE-15 received an insufficient rating for structural validity due to the inadequate design of the underlying studies [16]. Similarly, the SCC showed insufficient structural validity. No data on structural validity could be obtained for the German PEQ neither in the original publication nor in additional studies [25]. The same was true for the Danish National Cancer Questionnaire. PEACS and HCAHPS received a sufficient rating (Table 5). For the EORTC INPAT-SAT32 there is a metanalysis summarizing the data on structural validity [29]. There is a detailed analysis of structural validity for the PSCC-G [22], which shows mixed results.

**Table 5:** Overview of the analyses results on structural validity of German-language PREMs.

			1	2	3a	3b	4	5	6
		Reference	Is the PREM based on a reflective or formative model?	Does the study concern unidimensionality?	For CTT: Was exploratory or confirmatory factor analysis performed?	For IRT/Rasch: does the chosen model fit to the research question?	Was the sample size included in the analysis adequate?	Were there any other important flaws in the design or statistical methods of the study?	Overall rating on structural validity
**generic PREMs**		** **	** **						
**1**	NORPEQ	Oltedal S, et al. Scandinavian Journal of Public Health, 2007; 35: 540–547	reflective	no	very good (confirmatory and explorative)	N/A	very good (n = 244 participants for 8 questions)	very good	?*
**2**	Picker PPE-15	Jenkinson C, Coulter A, Bruster S. Int J Qual Health Care. 2002;14(5):6.	reflective	yes	inadequate	N/A	vey good	inadequate	-
**3**	PEACS	Noest S, Ludt S, Klingenberg A, et al., Int J Qual Health Care. 2014;26(3):10.	reflective	yes	very good (confirmatory and explorative)	N/A	very good (n = 492 participants for 59 questions)	very good	+
**4**	HCAHPS	Keller S, et al. Health Serv Res. 2005;40:2057–77.	reflective	yes	very good (confirmatory and explorative)	N/A	very good (n = 19,720 participants)	very good	+
**5**	QPPS	No data available on structural validity for the QPPS	reflective	0	0	N/A	0	0	?
	QPP	Larsson G, Larsson BW, Munck IME. Scand J Caring Sci. 1998;12:111–8.;	reflective	yes	very good (confirmatory)	N/A	very good (n = 611 participants)	very good	+
**6**	DUQUE	zum DUQUE sind keine Studien zur Strukturvalidität vorhanden	formative	0	0	N/A	0	0	?
**7**	PEQ (German)	zum PEQ (German) sind keine Studien zur Strukturvalidität vorhanden	reflective	0	0	N/A	0	0	?
**8**	Schoenfelder et al.	Tonio Schoenfelder, et al. International J. Quality in Health Care, Volume 23, Issue 5, October 2011, Pages 503–509	reflective	yes	adequate (explorative)	N/A	very good (n = 5,774 participants)	very goodt	?*
**cancer-care specific PREMs**									
**1**	EORTC IN-PATSAT32	Neijenhuijs KI, et al. Support Care Cancer. 2018 Aug;26(8):2551–2560.	reflective	yes	very good (confirmatory)	N/A	very good	inadequate	-
**2**	PSCC-G	Bokemeyer F, Lange-Drenth L, Jean-Pierre P, Schulz H, Bleich C. BMC Health Serv Res. 2020 Oct 27;20(1):983	reflective	yes	very good (confirmatory and explorative)	N/A	very good (n = 394 participants, 18 questions)	very good	+/-**
**3**	Danish National Cancer Patient Questionnaire	Rudolph C, Petersen GS, Pritzkuleit R, Storm H, Katalinic A. BMC Health Serv Res. 2019 Nov 1;19(1):786	reflective	0	0	N/A	0	0	?
		Danish Cancer Society. Kræftpatienters behov og oplevelser med sundhedsvæsenet under udredning og behandlingKræftens Bekæmpelses, 2017. ISBN: 978-87-7064-367-2	reflective	?	0	N/A	very good	0	
**4**	SCCC	Esser P, Sautier L, Sarkar S, Schilling G, Bokemeyer C, Koch U, Friedrich M, Defossez G, Mehnert-Theuerkauf A. Health Qual Life Outcomes. 2021 May 17;19(1):147	reflective	yes	adequate (explorative)	N/A	very good (n = 333 participants, 32 questions)	doubtful	-

“+” = suficient,”–”= insufficient, “?” = unclear

* not all necessary information for a "+" rating available.

** all data available, but mixed results

0 = not reported

N/A = not applicable

### Internal consistency

Table 6 shows the results of internal consistency studies of German-language PREMs. Cronbach´s Alpha for the NORPEQ (containing only one scale) is 0.85 [20]. A single validation study is available for the PPE-15 [16]. It is unclear whether the consistency statistics contained in this study are calculations of Cronbach´s Alpha or not. Furthermore, the consistency analyses are not available for the various PPE-15 subscales, which are partly dichotomous, partly continuous. Consequently, the PPE-15 received an “insufficient” rating for study design. Similarly, internal consistency analyses (Cronbach`s Alpha) of the PEQ refer to the entire questionnaire and not to the various subscales [25]. There are no internal consistency studies for the PEACS questionnaire. Keller et al. reported data on internal consistency for all 7 subscales of the HCAHPS [19]. With exception of the subscale “*Medicine Communication*”(Cronbach`s Alpha 0.66) all HCAHPS subscales show a Cronbach`s Alpha ≥0.70. Analysis of internal consistency is available for all subscales of the QPPS in the study by Larsson et al. [18]. Results are mixed as some subscales exhibit Cronbach`s alpha ≥0.70 and some <0.70. No data on internal consistency could be found for the DUQUE and the *Danish National Cancer Patient Questionnaire*. For the PREM by Schoenfelder et al. internal consistency results are available for both subscales of the questionnaire (*received service* and *medical aspects)*. Both of which have a Cronbach´s Alpha ≥0.70.

**Table 6:** Overview of internal consistency studies of German-language PREMs. Rating according to COSMIN guidelines.

			1	2	3		4
		Reference	Does the scale consist of effect indicators, i.e., is it based on a reflective model	Was an internal consistency statistic calculated for each unidimensional scale or subscale separately?	For continuous scores: Was Cronbach’s alpha or omega calculated?	For dichotomous scores: Was Cronbach’s alpha or KR‐20 calculated?	Overall rating
**generic PREMs**							
**1**	NORPEQ	Oltedal S, et al. Scandinavian Journal of Public Health, 2007; 35: 540–547	reflective	very good (contains only one scale)	very good (calculation of Cronbach’s Alpha)	N/A	**+**
**2**	Picker PPE-15	Jenkinson C, et al. Int J Qual Health Care. 2002;14(5):6.	reflective	insufficient (see text)	very good (calculation of Cronbach’s Alpha)	insufficient	**?**
**3**	PEACS	Noest S, et al., Int J Qual Health Care. 2014;26(3):10.	reflective	0	0	0	**?**
**4**	HCAHPS	Keller S, et al. Health Serv Res. 2005;40:2057–77.	reflective	very good (calculation for each subscale)	very good (calculation of Cronbach’s Alpha)	N/A	**+/-**
**5**	QPPS	Larsson BW, Larsson G. J Clin Nurs. 2002;11:681–7.	reflective	very good (calculation for each subscale)	very good (calculation of Cronbach’s Alpha)	N/A	**+/-** **
**6**	DUQUE	No studies could be identified	formative	0	0	0	**?**
**7**	PEQ (German)	No studies could be identified	reflective	insufficient (see text)	very good (calculation of Cronbach’s Alpha)	N/A	**?**
**8**	Schoenfelder et al.	Tonio Schoenfelder, et al. Journal for Quality in Health Care, Volume 23, Issue 5, October 2011, Pages 503–509	reflective	very good (calculation for each subscale)	very good (calculation of Cronbach’s Alpha)	N/A	**+**
**cancer-care specific PREMs**							
**1**	EORTC IN-PATSAT32	Neijenhuijs KI, et al. Support Care Cancer. 2018 Aug;26(8):2551–2560.	reflective	very good (calculation for each subscale)	very good (calculation of Cronbach’s Alpha)	N/A	**+/-**
**2**	PSCC-G	Bokemeyer F, et al. BMC Health Serv Res. 2020 Oct 27;20(1):983	reflective	sufficient	very good (calculation of Cronbach’s Alpha)	N/A	**+**
	PSCC (engl. Original version)	Jean-Pierre P, et al, Group PNRP. Cancer. 2011;117(4):854–61	reflective	sufficient	very good (calculation of Cronbach’s Alpha)	N/A	**+**
	RESPERES (subscale "Information"; part of the German PSCC-G)	Defossez G, et al. Health Qual Life Outcomes. 2021 May 17;19(1):147	reflective	sufficient	very good (calculation of Cronbach’s Alpha)	N/A	**+**
	PASQOC (subscale "family and friends", "shared-decision making" & "nursing" part of the German PSCC-G)	Kleeberg UR, et al. M. Support Care Cancer. 2008;16(8):947–54.	reflective	sufficient	very good (calculation of Cronbach’s Alpha)	N/A	**+**
**3**	Danish National Cancer Patient Questionnaire	Rudolph C, et al. BMC Health Serv Res. 2019 Nov 1;19(1):786	reflective	0	0	0	**?**
		Danish Cancer Society. Kræftpatienters behov og oplevelser med sundhedsvæsenet under udredning og behandlingKræftens Bekæmpelses, 2017. ISBN: 978-87-7064-367-2	reflective	0	0	0	**?**
**4**	SCCC	Esser P, et al. Health Qual Life Outcomes. 2021 May 17;19(1):147	reflective	insufficient	very good (calculation of Cronbach’s Alpha)	N/A	**+**

“+” = sufficient,”–”= insufficient, “?” = unclear

* not all information available for "+" rating.

** all data available, but mixed results (see text)

0 = not reported

N/A = not applicable

The internal consistency of the EORTC IN-PATSAT32 has been analyzed in a recent metaanalysis [29]. According to this metaanalysis, 5 studies are available on the internal consistency of the IN-PATSAT32, of which 3, however, are of poor methodological quality. Analyses are available for all subscales of the questionnaire, with Cronbach`s Alpha ≥0.70 across all scales, except for the subscale *hospital access* (Cronbach`s Alpha <0.70). The overall Cronbach´s Alpha for the entire PSCC-G is reported to be 0.92 [22]. As the German version of the PSCC (PSCC-G) consists of the German translation of the English PSCC [30] as well as of translations of parts of the French REPERES (subscale „Information“) [31] and German PASQOC [32] (subscales *family and friends*, *shared-decision making* and *nursing staff*), the original questionnaires can be analyzed for internal consistency as well. For all subscales Cronbach´s Alpha is reported to be ≥0.70.

### Measurement invariance / cross-cultural validity

“Cross-cultural validity refers to the degree to which the performance of the items on a translated or culturally adapted instrument are an adequate reflection of the performance of the items of the original version of the instrument” [11]. It is particularly important for PREM translations into German. Results are depicted in Table 7. No data on cross-cultural validity could be found for the DUQUE questionnaire. A cross-cultural validation study for the NORPEQ is available is available for several Scandinavian countries, but not for German. The PPE-15 underwent validation in 5 countries including German speaking countries (Switzerland and Germany) [16]. The patient cohorts were comparable in respect to sex and age, but not in respect to indications (elective vs. emergency). No further details about the patient cohorts were collected. Furthermore, no adequate study could be identified that compares the English original questionnaire with the German translation [16]. Such a study exists for the Spanish language [33]. This study shows that an expansion of the questionnaire from 15 to 33 questions (PPE-33) is necessary to preserve psychometric properties [33]. There is a cross-cultural validation study for the HCAHPS PREM [34]. In this study it is unclear whether patient cohorts were comparable. Furthermore, only 10 German-speaking patients were included [34]. In addition, the statistical methods used are considered to be inadequate according to current COSMIN guidelines. The QPPS has been used in two German studies without prior validation of the German translation [28, 35]. Validation of the EORTC IN-PATSAT32 occurred in an international study in which 34 German-speaking patients were included [36]. However, cross-cultural validity was not analyzed in this study. An addition, the EORTC IN-PATSAT32 metaanalysis by Neijenhuijs et al. found no studies on cross-cultural validation of the questionnaire in German [29]. Similarly, no cross-cultural validation in German could be found for the Danish *National Cancer Questionnaire* [37]. Rudolph et al. use the Danish *National Cancer Questionnaire* in their study but without previous validation [21]. The PSCC-G has been validated in German in a study by Bokemeyer et al. with a sufficient number of patients and adequate statistical methods [22]. The remaining PREMs (PEACS, PEQ, Schoenfelder et al, SCCC) have been developed in German including patients from different medical specialities.

**Table 7:** Results of cross-cultural validity analyses of PREMs into German. Rating according to COSMIN guidelines.

			Reference	Were the samples similar for relevant characteristics except for the group variable?	Was an appropriate approach used to analyse the data?	Was the sample size included in the analysis adequate?	Overall rating
**generic PREMs**							
**1**	NORPEQ		Oltedal S, et al. Scandinavian Journal of Public Health, 2007; 35: 540–547	0	0	0	?
**2**	Picker PPE-15		Jenkinson C, Coulter A, Bruster S. Int J Qual Health Care. 2002;14(5):6.	doubtful	inadequate	adequate	?
**3**	PEACS		Noest S, Ludt S, Klingenberg A, et al., Int J Qual Health Care. 2014;26(3):10.	N/A*	N/A*	N/A*	N/A*
**4**	HCAHPS	Squire et al. International Journal for Quality in Health Care 2012; Volume 24, Number 5: pp. 470–475		doubtful	doubtful	inadequate	?
**5**	QPPS	Singer S, et al. Langenbecks Arch Surg. 2009, 394:723–731		0	0	0	?
		Singer S, Danker H, et al. J Cancer Res Clin Oncol. 2019		0	0	0	
**6**	DUQUE	no studies on measurement invariance / cross-cultural validity could be found		0	0	0	?
**7**	PEQ (German)	Christoph Gehrlach, et al. Verlag Bertelsmann Stiftung. ISBN: 978-3-86793-021-5. 2009.		N/A*	N/A*	N/A*	N/A*
**8**	Schoenfelder et al.	Tonio Schoenfelder, Joerg Klewer, Joachim Kugle. International Journal for Quality in Health Care, Volume 23, Issue 5, October 2011, Pages 503–509		N/A*	N/A*	N/A*	N/A*
**cancer-care specific PREMs**							
**1**	EORTC IN-PATSAT32	Neijenhuijs KI, Jansen F, et al. Support Care Cancer. 2018 Aug;26(8):2551–2560.		0	0	inadequate	?
		A. Brédart, A. Bottomley, Jet al. European Journal of Cancer 41 (2005) 2120–2131		0	0	inadequate	N/A
**2**	PSCC-G	Bokemeyer F, Lange-Drenth L, Jean-Pierre P, Schulz H, Bleich C. BMC Health Serv Res. 2020 Oct 27;20(1):983		adequate	very good	very good	+
**3**	Danish National Cancer Patient Questionnaire	Rudolph C, et al. BMC Health Serv Res. 2019 Nov 1;19(1):786		0	0	0	?
		Danish Cancer Society. Kræftpatienters behov og oplevelser med sundhedsvæsenet under udredning og behandlingKræftens Bekæmpelses, 2017. ISBN: 978-87-7064-367-2		0	0	0	?
**4**	SCCC	Esser P, et al. Health Qual Life Outcomes. 2021 May 17;19(1):147		N/A*	N/A*	N/A*	N/A*

“+” = sufficient,”–”= insufficient, “?” = unclear

N/A: not applicable as the PREM has been developed in German.

0: not reported

### Reliability

An overview of the results of the reliability analyses can be found in Table 8. A study measured test-retest reliability of the NORPEQ [20] for which 68 of 244 patients were resent the questionnaire within 5–6 days. The intraclass correlation index (ICC) was between 0.45 (*nurse professional skills*) and 0,83 (*doctors understandable*). Four of the eight subscales exhibited an ICC <0.70. The test-retest reliability for the total score was 0.88 [20]. Consequently, the overall reliability rating for the NORPEQ was +/-. Noest et al. analyzed reliability for the PEACS questionnaire [24]. Test-retest reliability was measured via weighted kappa. With exception of the subscale institutional treatment and transition (weighted kappa 0,671), the weighted kappa was ≥0.70 for all subscales. Thus, overall rating was +. The study by Keller et al. analyzed hospital-level reliability [19]. No data on test-retest reliability could be identified for the HCAHPS. Hospital-level reliability assumes that recurrent measurements (retesting) of patients in the same hospital should be more similar than recurrent measurements in another hospital. In the study by Keller et al. 300 patients from different hospitals were asked to fill out the HCAHPS. For six of the seven subscales the ICC value was ≥0.70. The only exception was *medicine communication* (ICC 0,66).

**Table 8:** Results of reliability analyses of German-language PREMs. Rating according to COSMIN guidelines.

			1	2	3	4	5	6	7	8	
		Reference	Were patients stable in the interim period on the construct to be measured?	Was the time interval appropriate?	Were the test conditions similar for the measurements?	For continuous scores: Was an intraclass correlation coefficient (ICC) calculated?	For dichotomous/nominal/ ordinal scores: Was kappa calculated?	For ordinal scores: Was a weighted kappa calculated?	For ordinal scores: Was the weighting scheme described? e.g. linear, quadratic	Were there any other important flaws in the design or statistical methods of the study?	Overall rating
**generic PREMs**											
**1**	NORPEQ	Oltedal S, et al. Scandinavian Journal of Public Health, 2007; 35: 540–547	very good	very good (5–6 days)	very good	adequate	N/A	N/A	N/A	very good	+/-
**2**	Picker PPE-15	Jenkinson C, Coulter A, Bruster S. Int J Qual Health Care. 2002;14(5):6.	0	0	0	0	N/A	N/A	N/A	0	?
**3**	PEACS	Noest S, et al., Int J Qual Health Care. 2014;26(3):10.	very good	very good (3 weeks)	very good	N/A	very good	very good	N/A	very good	+
**4**	HCAHPS	Keller S, et al. Health Serv Res. 2005;40:2057–77.	doubtful	N/A*	adequate	adequate	N/A	N/A	N/A	very good	+
**5**	QPPS	No reliability studies for the QPPS or QPP could be identified	0	0	0	0	N/A	N/A	N/A	0	?
**6**	DUQUE	No reliability studies for the DUQUE could be identified	0	0	0	0	N/A	N/A	N/A	0	?
**7**	PEQ-G	Christoph Gehrlach, Thomas Altenhöner, David Schwappach. Verlag Bertelsmann Stiftung. ISBN: 978-3-86793-021-5. 2009.	doubtful	N/A*	doubtful	inadequate	N/A	N/A	N/A	?	-
**8**	Schoenfelder et al.	Tonio Schoenfelder, et al. Int.J for Quality in Health Care, Volume 23, 5, 2011, 503–509	0	0	0	0	N/A	N/A	N/A	0	?
**cancer-care specific PREMs**											
**1**	EORTC IN-PATSAT32	Obtel, M. et al. Asian Pac J Cancer Prev 18, 1403–1409 (2017).	very good	inadequate	very good	adequate	N/A	N/A	N/A	doubtful	+
		Pishkuhi, M. A., et al. Asian Pac J Cancer Prev 15, 10121–10128 (2014).	very good	adequate	very good	doubtful	N/A	N/A	N/A	doubtful	
**2**	PSCC-G	No reliability studies for the PSCC-G could be identified	0	0	0	0	N/A	N/A	N/A	0	?
	PSCC (engl. original)	No reliability studies for the PSCC could be identified	0	0	0	0	N/A	N/A	N/A	0	?
**3**	Danish National Cancer Patient Questionnaire	No reliability studies for the could be identified	0	0	0	0	N/A	N/A	N/A	0	?
**4**	SCCC	No reliability studies for the SCCC could be identified	0	0	0	0	N/A	N/A	N/A	0	?

“+” = sufficient,”–”= insufficient, “?” = unclear

N/A: not applicable

0: not reported

* for the HCAHPS and the PEG (German) studies measured “hospital level realibility”, no data on test-retest, inter-rater or intra-Rater reliability

The overall rating for the HCAHPS was +. A similar hospital-level reliability analysis can be found for the PEQ in the study by Gehrlach et al [25]. Logistic regression analyses were used to find out whether the questionnaire can distinguish between patient cohorts from different hospitals. However, no specific results are reported except for “…none of the instruments showed significant results”. Further reliability data for the PEQ could not be identified. No reliability study could be identified for the PPE-15, QPPS or the QPP, DUQUE, PSCC-G, the Danish National Cancer Patient Questionnaire or the SCCC.

For the cancer-care specific PREM EORTC IN-PATSAT32 two studies investigate test-retest reliability [38, 39]. Appreciation of reliability in these two studies has already been done by Neijenhuijs et al. [29]. ICC was ≥0.70 for all subscales of the EORTC IN-PATSAT32 in the study by Pishkuhi et al. In the study by Obtel et al. all scales except *doctor availability* (correlation coefficient 0.64) and *overall satisfaction* (correlation coefficient 0.67) showed a correlation coefficient ≥0.70. Consequently, overall reliability rating was +. However, both studies showed methodological weaknesses as the time interval between test and retest was too short (30 minutes) [39] or it was unclear, which type of correlation coefficient has been used [38].

### Analysis of measurement error

An overview of the results for the analysis of measurement error can be found in Table 9. Measurement error could not be analyzed as *no minimal important change* has been defined for any of the German PREMs so far. Therefore, all PREMs received “?” rating. Only for HCAHPS there has been a calculation of the standard error of Measurement (SEM). For the EORTC IN-PATSAT32 the SEM and SDC can be calculated from the studies by Obtel et al. [39] and Pishkuhi et al. [38] as has been shown by Neijenhuijs et al. [29]. However, as no *minimal important change* has been defined for the EORTC IN-PATSAT32 an overall rating of the measurement error is not possible.

**Table 9:** Results of analyses for measurement error of German-language PREMs. Rating according to COSMIN guidelines.

			1	2	3	4	5	6
		Reference	Were patients stable in the interim period on the construct to be measured?	Was the time interval appropriate?	Were the test conditions similar for the measurements?	For continuous scores: Was the Standard Error of Measurement (SEM), Smallest Detectable Change (SDC) or Limits of Agreement (LoA) calculated?	For dichotomous/ nominal/ ordinal scores: Was the percentage (positive and negative) agreement calculated?	Overall rating
**generic PREMs**															
**1**	NORPEQ	Oltedal S, et al. Scandinavian Journal of Public Health, 2007; 35: 540–547	very good	very good (5–6 days)	very good	0	0	?
**2**	Picker PPE-15	Jenkinson C, Coulter A, Bruster S. Int J Qual Health Care. 2002;14(5):6.	0	0	0	0	0	?
**3**	PEACS	Noest S, et al., Int J Qual Health Care. 2014;26(3):10.	very good	very good (3 weeks)	very good	0	0	?
**4**	HCAHPS	Keller S, et al. Health Serv Res. 2005;40:2057–77.	doubtful	N/A*	adequate	very good	N/A	?
**5**	QPPS	No studies on measurement error were identified	0	0	0	0	0	?
**6**	DUQUE	No studies on measurement error were identified	0	0	0	0	0	?
**7**	PEQ (German)	Christoph Gehrlach, et al. Verlag Bertelsmann Stiftung. ISBN: 978-3-86793-021-5. 2009.	doubtful	N/A*	doubtful	0	0	?
**8**	Schoenfelder et al.	No studies on measurement error were identified	0	0	0	0	0	?
**cancer-care specific PREMs**															
**1**	EORTC IN-PATSAT32	Obtel, M. et al. Asian Pac J Cancer Prev 18, 1403–1409 (2017).	very good	inadequate	very good	adequate	N/A				**?**
		Pishkuhi, M. A., et al. Asian Pac J Cancer Prev 15, 10121–10128 (2014).	very good	adequate	very good	adequate	N/A				
**2**	PSCC-G	No studies on measurement error were identified	0	0	0	0	0				**?**
**3**	Danish National Cancer Patient Questionnaire	No studies on measurement error were identified	0	0	0	0	0				**?**
**4**	SCCC	No studies on measurement error were identified	0	0	0	0	0				**?**

“+” = sufficient,”–”= insufficient, “?” = unclear

N/A: not applicable

0: not reported

### Analysis of criterion validity

As no gold standard for the measurement of patient-centeredness has yet been defined, overall criterion validity cannot be analyzed for PREMs. However, for some PREM subscales, gold standards for measurement are available. Consequently, criterion validity for these subscales can be analyzed. No data on criterion validity was found for NORPEQ, PPE-15, HCAHPS, QPPS (or QPP), DUQUE, Schoenfelder et al., EORTC IN-PATSAT-32, PSCC, SCC and the Danish National Cancer Patient Questionnaire.

The PEACS subscales *shared decision making* and *information at discharge* were compared to the two validated and established German questionnaires *Shared Decision Making Questionnaire* (SDM-Q-9) [40] and the *Care-Transition-Measurement* Questionnaire (CTM) [41], respectively. Both subscales showed a very high (SDM-Q-9; r = 0.814, p < 0.001) or high correlation (CTM-3; r = 0.511, p<0.001) with the respective gold standard [24].

The PEQ was correlated to the Cologne Patient Questionnaire *(Kölner Patientenfragebogen*, *KPF)*. For the PEQ subscales “physicians” and “nursing” there was a high correlation >0.70 with the KPF. Only a weak correlation was found for the subscale “management” (between -0,28 and -0,46) [25].

### Analysis of hypothesis testing for construct validity

Hypothesis testing for construct validity describes the degree to which a PREM results is consistent with an *a priori* hypothesis. The hypothesis to be tested can either be a comparison of PREM results between two clinically defined patient groups (*known groups validity)* or PREM subscales can be compared to another known measurement tool *(convergent validity)*. No data on hypothesis testing for construct validity could be found for: PEACS, DUQUE, PEQ-G, *Danish National Cancer Patient Questionnaire*. Results for all other German PREMs can be found in Table 10. Most hypotheses are tested positive, i.e., results confirm the a priori formulated hypothesis. However, all PREMs, except for the PSCC-G, also show negative results. For the PSCC-G only positive test results could be found.

**Table 10:** Results of hypothesis testing for construct validity of German-language PREMs. Rating according to COSMIN guidelines.

				Convergent validity			Known-groups validity			
		Reference	Hypothesis	Is it clear what the comparator instrument measures?	Were the measurement properties of the comparator instrument(s) sufficient?	Were design and statistical methods adequate for the hypotheses to be tested?	Was an adequate description provided of important characteristics of the subgroups?	Were design and statistical methods adequate for the hypotheses to be tested?	Result	Overall rating
**generic PREMs**										
**1**	NORPEQ	Oltedal S, et al. Scandinavian Journal of Public Health, 2007; 35: 540–547	A higher general satisfaction correlates positively with NORPEQ results	very good	very good	adequate	0	0	There is a correlation between NORPEQ scores and general satisfaction	+
		Oltedal S, et al. Scandinavian Journal of Public Health, 2007; 35: 540–547	Errors in care result in lower NORPEQ scores	very good	very good	adequate	0	0	There is a weak correlation between errors in care and NORPEQ results	+
		Oltedal S, et al. Scandinavian Journal of Public Health, 2007; 35: 540–547	Patients whose expectations have been met have higher NORPEQ scores	very good	very good	adequate	0	0	Results show the expected correlation	+
		Oltedal S, et al. Scandinavian Journal of Public Health, 2007; 35: 540–547	Patients with a higher general health status have higher NORPEQ scores	very good	very good	adequate	0	0	Results show the expected correlation	+
		Oltedal S, et al. Scandinavian Journal of Public Health, 2007; 35: 540–547	Age correlates positively with NORPEQ scores	0	0	0	very good	adequate	Results show the expected correlation	+
		Boge RM, Haugen AS, Nilsen RM, Bruvik F, Harthug S. BMJ Open Qual. 2019 Dec 16;8(4):e000728.	Patients that receive a discharge interview have higher NORPEQ scores	0	0	0	very good	inadequate	Results show a statistically significant difference. The correlation, however, is unclear	?
**1**	NORPEQ	Groene O, et al. PLoS One. 2015 Jul 7;10(7):e0131805.	Hospital quality initiatives increase the patient experience measured via the NORPEQ	0	0	0	very good	adequate	The expected correlation could not be found	-
		Leonardsen AL, et al. LP. BMC Health Serv Res. 2017 Sep 29;17(1):685.	Results of NORPEQ items correlate with comparable items of the PPE-15	very good	very good	very good	0	0	Results show a weak correlation between NORPEQ and PPE-15 items	+/-
**2**	Picker PPE-15	Harrison R, et al. Int J Qual Health Care. 2018 Jun 1;30(5):358–365.	Patients with an adverse event have higher PPE-15 scores	0	0	0	very good	adequate	Results show the expected correlation	+
		Leonardsen AL, et al. LP. BMC Health Serv Res. 2017 Sep 29;17(1):685.	Results of NORPEQ items correlate with comparable items of the PPE-15	very good	very good	very good	0	0	Results show a weak correlation between NORPEQ and PPE-15 items	+/-
		Wong EL, Leung MC, Cheung AW, Yam CH, Yeoh EK, Griffiths S. Int J Qual Health Care. 2011 Aug;23(4):390–6.	Patients in private hospitals in Hong-Kong have a better patient experience than patients in public hospitals	0	0	0	very good	very good	Results do not show higher PPE-15 scores in private hospitals	-
		Ashton F, Hamid K, Sulieman S, Eardley W, Baker P. Injury. 2017 Apr;48(4):960–965.	Patients whose surgery was postponed have lower PPE-15 scores	0	0	0	very good	inadequate	Results confirm the hypothesis, but no correlations were measured	?
		Ashton F, Hamid K, Sulieman S, Eardley W, Baker P. Injury. 2017 Apr;48(4):960–965.	Patients waiting >3 days for their operation have a lower score than patients waiting <3days	0	0	0	very good	inadequate	Results confirm the hypothesis, but no correlations were measured	?
**2**	Picker PPE-15	Ashton F, Hamid K, Sulieman S, Eardley W, Baker P. Injury. 2017 Apr;48(4):960–965.	Patients undergoing arthroscopy have a higher score than patients undergoing ankle fracture surgery	0	0	0	very good	inadequate	Results confirm the hypothesis, but no correlations were measured	?
		Murrells T, Robert G, Adams M, Morrow E, Maben J. BMJ Open. 2013 Jan 30;3(1):e002211.	Results of the PPE-15 subscales correlate negatively with results of the Patient Evaluation of Emotional Care during Hospitalisation’ (PEECH) subscales	very good	doubtful	adequate	0	0	Results show the expected negative correlation	+
		Andres EB, Song W, Song W, Johnston JM. BMC Health Serv Res. 2019 Sep 3;19(1):623.	Accredited hospitals have higher scores than non-accredited hospitals	0	0	0	very good	adequate	Results show the expected correlation	+
		Wolf A, Olsson LE, Taft C, Swedberg K, Ekman I. BMC Nurs. 2012 Jun 14;11:8.	Vulnerable patients have lower scores than non-vulnerable patients	0	0	0	very good	doubtful	Results show the expected correlation, however, groups differed in important aspects	+
**3**	PEACS	No studies on hypothesis testing for construct validity could be identified	0	0	0	0	0	0	0	?
**4**	HCAHPS	Keller S, O’Malley A, et al. Bull Hosp Jt Dis (2013). 2019 Dec;77(4):263–268.	Patients undergoing day-clinic surgery for hip-TEP have better scores than in-hospital patients undergoing the same surgery	0	0	0	very good	inadequate	Results show the expected correlation	+
		Huppertz JW, Smith R. J Healthc Manag. 2014 Jan-Feb;59(1):31–47.	positive and negative handwritten comments on hospital feedback questionnaires correlate with positive or negative HCHAPS results respectively	0	0	0	very good	inadequate	Results show the expected correlation	+
**4**	HCAHPS	Velez VJ, Kaw R, Hu B, Frankel RM, Windover AK, Bokar D, Rish JM, Rothberg MB. J Hosp Med. 2017 Jun;12(6):421–427.	The HCAHPS subscale “doctors communication” correlates with results of the Four Habits Coding Scheme (4HCS)	very good	very good	very good	0	0	Results did not show the expected correlation	-
		Huppertz JW, Otto P. Health Care Manage Rev. 2018 Oct/Dec;43(4):359–367.	HCAHPS scores correlate with social media comments	0	0	0	doubtful	adequate	Results show the expected correlation	+
		Bobrovitz N, et al. J Trauma Acute Care Surg. 2016 Jan;80(1):111–8.	For trauma patients HCAHPS scores correlate with results of the Quality of Trauma Care Patient-Reported Experience Measure (QTAC-PREM)	inadequate	doubtful	very good	0	0	Results show the expected correlation	+
		Joseph B, et al. J Trauma Acute Care Surg. 2017 Apr;82(4):722–727.	HACHPS scores correlate with clinical outcome parameters (complication scores, readmission, failure-to-rescue)	very good	N/A	very good	0	0	Results did not show the expected correlation	-
		Day MS, et al. J Healthc Qual. 2014 Nov-Dec;36(6):33–40.	Hospital-acquired conditions correlate with negative HCAHPS scores in an orthopedic hospital	0	0	0	adequate	very good	Results did not show the expected correlation	-
		Gupta A, et al. J Pain Res. 2009 Nov 13;2:157–64.	Adequate analgesic management results in higher HCAHPS scores	0	0	0	adequate	very good	Results show the expected correlation	+
		Kemp KA, et al. BMJ Open. 2016 Jul 1;6(7):e011242.	A patient-safety indicator correlates negatively with the HCAHPS global score and certain subscores	0	0	0	very good	adequate	Results show the expected negative correlation	+
		Sodhi N, et al. J Arthroplasty. 2019 Nov;34(11):2573–2579.	Patients readmitted within 30 or 90 days have lower HCAHPS scores than patients that have not been readmitted	0	0	0	very good	doubtful	Results show the expected negative correlation	+
**5**	QPPS	No studies on hypothesis testing for construct validity could be identified	0	0	0	0	0	0	0	?
**6**	DUQUE	No studies on hypothesis testing for construct validity could be identified	0	0	0	0	0	0	0	?
**7**	PEQ-G	No studies on hypothesis testing for construct validity could be identified	0	0	0	0	0	0	0	?
**8**	Schoenfelder et al.	Tonio Schoenfelder, et al. Int.J for Quality in Health Care, Volume 23, 5, 2011, 503–509	Higher age correlates inversely with the score	0	0	0	very good	very good	Results do not show the expected correlation	-
**cancer-care specific PREMs**										
**1**	EORTC IN-PATSAT32	Arraras JI, et al. Clin Transl Oncol. 2009 Apr;11(4):237–42.	Results of the IN-PATSAT32 correlate with results of the Oberst patients’ perception of care quality and satisfaction scale	very good	adequate	doubtful	0	0	Results show the expected correlation	+
		Arraras JI, et al. Clin Transl Oncol. 2009 Apr;11(4):237–42.	Results of the IN-PATSAT32 correlate with results of the EORTC QLQ-INFO25	very good	very good	doubtful	0	0	Results show the expected correlation	+
**1**	EORTC IN-PATSAT32	Arraras JI, et al. Clin Transl Oncol. 2009 Apr;11(4):237–42.	Patients with higher Oberst “perception of care quality and satisfaction scale” scores have higher IN-PATSAT32 scores	0	0	0	very good	doubtful	Results show the expected correlation	+
		Arraras JI, et al. Clin Transl Oncol. 2009 Apr;11(4):237–42.	Patients rating “would recommend the hospital to others” higher, show higher global IN-PATSAT32 scores	0	0	0	very good	doubtful	Results show the expected correlation	+
		Zhang J, Xie S, Liu J, Sun W, Guo H, Hu Y, Gao X. Patient Prefer Adherence. 2014 Sep 18;8:1285–92.	Patients <58 years have higher scores than patients ≥58 years	0	0	0	very good	inadequate	Results show the expected correlation	?
		Zhang J, Xie S, Liu J, Sun W, Guo H, Hu Y, Gao X. Patient Prefer Adherence. 2014 Sep 18;8:1285–92.	Patients with lower educational standard give lower IN-PATSAT32 scores	0	0	0	adequate	inadequate	Results show the expected correlation	?
		Zhang L, Dai Z, Cheng S, et al. Support Care Cancer. 2015 Sep;23(9):2721–30.	Patients with lower educational standard give lower IN-PATSAT32 scores	0	0	0	adequate	inadequate	Results show the expected correlation	?
		Zhang L, Dai Z, Cheng S, et al. Support Care Cancer. 2015 Sep;23(9):2721–30.	Patients waiting >2 months for diagnostic procedures have lower scores than patients waiting <2monts	0	0	0	very good	inadequate	The expected correlation was not found	?
		Asadi-Lari M, et al. Support Care Cancer. 2015 Jul;23(7):1875–82.	Results of the IN-PATSAT32 correlate with results of the EORTC QLQ-INFO25	very good	very good	inadequate	0	0	Results show the expected correlation	+
		Aboshaiqah A, et al. Palliat Support Care. 2016 Dec;14(6):621–627.	Results of the IN-PATSAT32 correlate with results of the QOLQ-C15-PAL	very good	adequate	inadequate	0	0	Results correlate with physical function, emotional function und dem global health status	?
**2**	PSCC-G	Bokemeyer F, et al. BMC Health Serv Res. 2020 Oct 27;20(1):983	Higher scores in the REPERES subscale “patient satisfaction” correlate with higher PSCC-G scores	very good	very good	doubtful	0	0	Results show the expected correlation	+
		Bokemeyer F, et al. BMC Health Serv Res. 2020 Oct 27;20(1):983	Higher scores in the PASCOQ subscale “family and friends” correlate with higher PSCC-G scores	very good	very good	doubtful	0	0	Results show the expected correlation	+
		Bokemeyer F, et al. BMC Health Serv Res. 2020 Oct 27;20(1):983	Higher scores in the PASCOQ subscale “shared-decision making” correlate with higher PSCC-G scores	very good	very good	doubtful	0	0	Results show the expected correlation	+
		Bokemeyer F, et al. BMC Health Serv Res. 2020 Oct 27;20(1):983	Higher scores in the PASCOQ subscale “nursing and care” correlate with higher PSCC-G scores	very good	very good	doubtful	0	0	Results show the expected correlation	+
		Jean-Pierre P, Fiscella K, et al. Cancer. 2011;117(4):854–61	Higher results in the subscale “understanding and participation in care" of the CASE Cancer questionnaire correlate with higher PSCC scores	very good	very good	doubtful	0	0	Results show the expected correlation	+
		Jean-Pierre P, Fiscella K, et al. Cancer. 2011;117(4):854–61	Higher results in the subscale “seek and obtain information" of the CASE Cancer questionnaire correlate with higher PSCC scores	very good	very good	doubtful	0	0	Results show the expected correlation	+
**2**	PSCC-G	Jean-Pierre P, Fiscella K, et al. Cancer. 2011;117(4):854–61	Age, sex, and native language do not correlate with PSCC-G scores	0	0	0	very good	doubtful	Results show the expected lack of correlation	+
**3**	Danish National Cancer Patient Questionnaire	No studies on hypothesis testing for construct validity could be identified	0	0	0	0	0	0	0	?
**4**	SCCC	Esser P, et al. Health Qual Life Outcomes. 2021 May 17;19(1):147	Higher scores in the depression subscale correlate inversely with the global SCCC score	inadequate	inadequate	very good	0	0	Results show the expected negative correlation	+
		Esser P, et al. Health Qual Life Outcomes. 2021 May 17;19(1):147	Higher scores in the anxiety subscale correlate inversely with the global SCCC score	inadequate	inadequate	very good	0	0	Results show the expected negative correlation	+
		Esser P, et al. Health Qual Life Outcomes. 2021 May 17;19(1):147	Higher scores in the fatigue subscale correlate inversely with the global SCCC score	inadequate	inadequate	very good	0	0	Results show the expected negative correlation	+
		Esser P, et al. Health Qual Life Outcomes. 2021 May 17;19(1):147	Higher scores in the “total symptom load” subscale correlate inversely with the global SCCC score	inadequate	inadequate	very good	0	0	Results show the expected negative correlation	+

“+” = sufficient,”–”= insufficient, “?” = unclear

N/A: not applicable

0: not reported

## Discussion

In the current study numerous German language PREMs could be identified that were not contained in previous publications [8, 42]. This was due to publications in recent years as well as due to the difficulty in identifying PREMs by database searches alone. Many German PREMs were found by hand-searches. The current study uses for the first time the current COSMIN guidelines for the assessment of PREMs [15]. Furthermore, by using a comprehensive framework of PC covering all dimensions of PC (S1 Table) a thorough analysis of content validity of PREMs was possible for the first time. The results show the lack of patient-relevant content domains in all 12 PREMs, not only for those developed in German, but also for commonly used international PREMs (Table 3). In addition, all included PREMS show deficits in the results or evaluation of psychometric measurement properties according to current COSMIN guidelines. Based on these results, context-specific application of German PREMs is mandatory and several recommendations can be made.

### Recommendations

**Fig 2:**
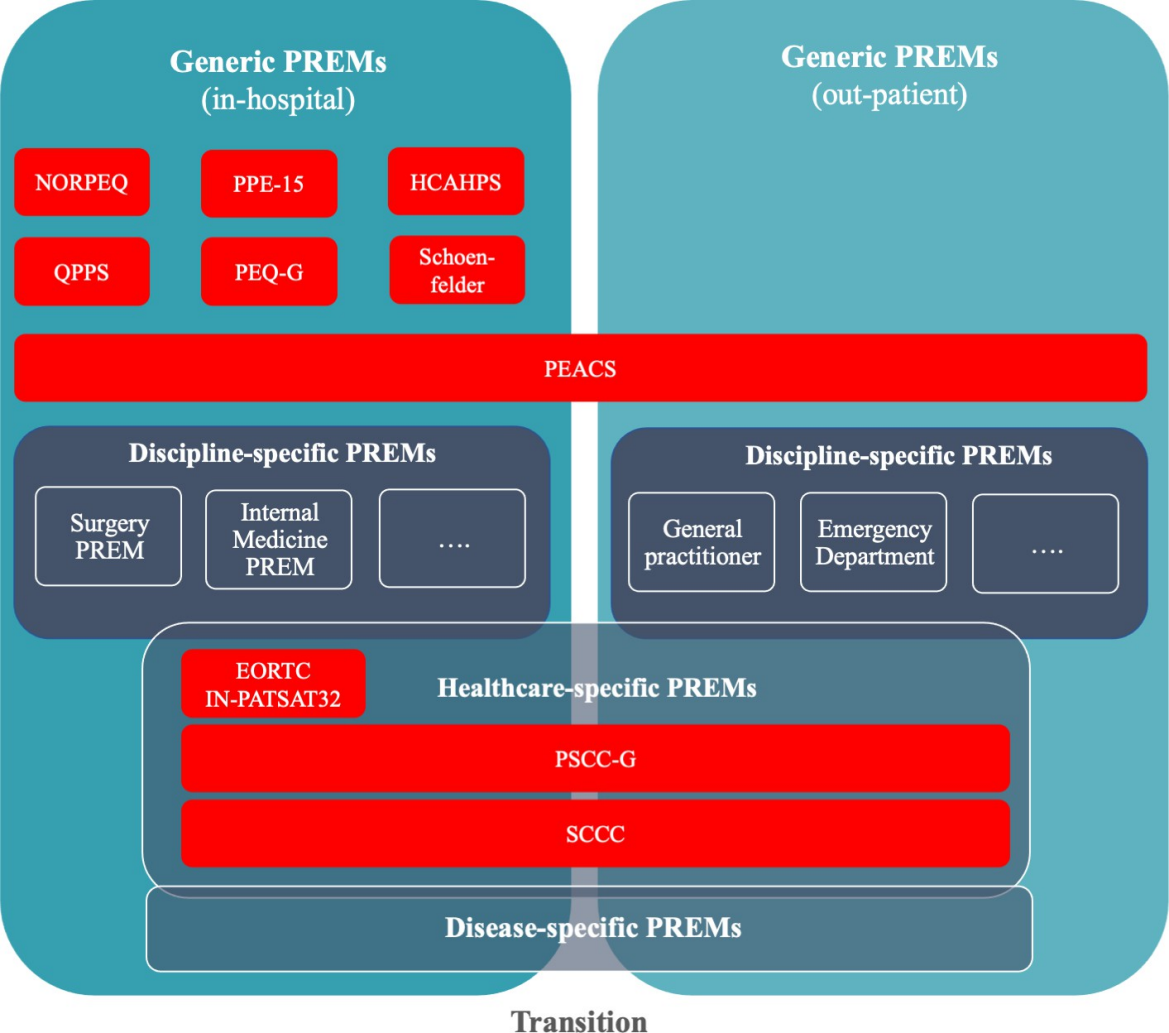
German language Patient-Reported Experience Measures (PREMs). Overview of German PREMs in their intended category of use.

Two out of the 12 PREMs cannot be recommended for use in German because of a lack of validation of psychometric properties: the DUQUE questionnaire [27], as well as the German translation of the *Danish National Cancer Patient Questionnaire* used by Rudolph et al. [21]. Depending on the intended use, one of the remaining ten PREMs can be selected. Fig 2 shows a schematic representation of the remaining PREMs within their intended area of use. The figure can facilitate preliminary PREM selection. In a next step, the results of this systematic review can be used to select a PREM with sufficient psychometric properties (Tables 5–10) and the necessary content (Tables 3 and 4). For example, for cancer care the PSCC-G has significant better psychometric properties than the SCC with its insufficient structural validity and lack of assessment in many psychometric domains. Furthermore, when selecting a PREM the intended area of application should to be considered (see introduction) [8]: is it intended as a reflection instrument for patients or rather as a provider-specific evaluation instrument for internal use or as a benchmarking instrument to compare different providers? In each case different content dimensions (Table 3) and length of questionnaires (Table 2) are of interest.

The following generic PREMs have been sufficiently evaluated in German: HCAHPS, NORPEQ, PPE-15 and PEACS. For cancer care the EORTC IN-PATSAT32 and PSCC-G have been adequately assessed and can currently be recommended. We were unable to identify a surgery-specific PREM in the German language. However, even for the above mentioned generic and cancer care-specific PREMs certain deficits need to be considered before use. The HCAHPS for example, although showing sufficient psychometric properties in many areas, exhibits deficits in its cross-cultural validation into German (Table 7) [34]. Some of its demographic questions like “What is your race? Please select at least one.” were rated poorly by native German speakers [34] and refer to its development in a different sociocultural context. Therefore, an adaptation to the German-speaking sociocultural context seems necessary. The PPE-15, one of the most frequently used PREMs worldwide, exhibits poor structural validity, while covering many dimensions of PC (sufficient content validity). In addition, for many psychometric properties of the PPE-15 no data could be found. We cannot rule out that such data exist but has not been published by the Picker institutes or was not identified by our search. The NORPEQ is an extensively studied PREM with adequate psychometric properties. However, cross-cultural validation studies only exist for languages other than German, although it has been used in a non-validated German translations [43]. One of the most extensively evaluated generic PREMs is the PEACS questionnaire, that has been developed in German with involvement of patients. It is a comprehensive questionnaire with more than 50 questions covering many aspects of PC. Because of its length (Table 2) its intended use is as a reflection instrument for patients and as an assessment tool for providers rather than as benchmarking instrument. It is the only German generic PREM that covers not only in-hospital aspects of patient experience, but also the transition into out-patient care. Although the PEACS has sufficient psychometric properties in many areas, there is a lack of data for test-retest, inter-rater and intra-rater reliability. The PSCC-G is a cancer care specific PREM, that covers transition aspects of care. It has been built from a validated German translation of an English questionnaire with additional questions from other languages (Table 2). The PSCC-G scored adequately in many psychometric domains, but data on test-retest, inter-rater and intra-rater reliability are lacking.

### Limitations

The study has several limitations. First, the search was limited to generic, surgery- and cancer care specific PREMs, i.e., PREMs for other disciplines (e.g., internal medicine) as well as PREMs for specific diseases were excluded. These PREMs can be found in the excluded fulltext list (S4 Table). Another limitation could have been the search algorithm. The fact, that many German-language PREMs were identified by hand-searching rather than the database search, could be a hint that the search algorithm was not specific enough. However, the large number of identified and screened articles indicates that our search was broad. Furthermore, we were able to identify significantly more German-language PREMs than in previous reviews [8, 42]. Identifying PREMs in scientific databases is not easy. Contrary to PROMs no PREM-specific taxonomy (e.g., MeSH term) exists for PREMs in common medical databases. As pointed out, patient centredness and patient experience are only beginning to be clearly defined (S1 Fig and S2 Table). The delineation to other concepts like patient satisfaction is not always clear cut which makes building a search algorithm more difficult.

A main finding of the study is the lack of psychometric data for many of the included PREMs. Frequently we were unable to find appropriate studies in accessible databases. However, many PREMs have been developed and are implemented by independent or commercial institutions or healthcare agencies. These institutions are often not scientifically driven and might not publish all available psychometric data. Exception are the transparent development and publication of data by the U.S. *Agency for Healthcare Research and Quality (AHRQ)* (www.ahrq.gov/cahps/about-cahps/index.html) or the Swedish PREMs [18, 44, 45].

### Future research

If PC is supposed to be more than a declaration of intent of healthcare politicians, it will require the implementation of PREMs into everyday clinical practice via the following measures:

- As shown in our study, there is no comprehensive modular PREM system in the German language comparable to other countries [23]. Many areas of healthcare (e.g., surgery) are not covered with available German-language PREMs. Consequently, the development, translation and testing of new PREMs is necessary.
- The missing psychometric properties of currently available German-language PREMs need to be evaluated.
- Most of the PREMs currently available are paper-based versions (Table 2). For broad implementation and timely assessment in hospitals and doctor´s offices electronic PREMs (ePREM) seem necessary. For this purpose, paper-based PREMs will need to be evaluated as digital versions and electronic systems will have to be developed and implemented that adhere to local data safety regulations. An integration into available hospital information systems is desirable, to facilitate the use in everyday clinical practice.
- It is unclear which conclusions should be drawn from the results of PREM (sub)scales. If providers adapt their service based on PREM results, there is little evidence-base to guide such changes [46]. Individualized local measures may be implemented, but there may also be standardized interventions, which can be tested in randomized-controlled trials which might improve aspects of PC and subsequently PREM results. More research is needed in this field.

There are two projects that should be mentioned in this context. First, the EORTC is currently developing and testing the PATSAT-33, a cancer-care specific PREM that will not only cover in-hospital patients, but also aspects of PC in out-patient settings as well as the transitional period [47]. A phase IV validation study in several European countries is underway including German-speaking countries. Second, the Hamburg-based ASPIRED project [48], is currently developing a German-language PREM, that will cover all aspects of PC according to Scholl et al. [5]. Both projects will close important evidence gaps.

## Conclusions

This is the first systematic review using a comprehensive framework of patient centredness and shows that none of the included PREMs, even those translated from other languages into German, cover all aspects of patient centredness. Furthermore, all included PREMS show deficits in the results or evaluation of psychometric measurement properties. Nonetheless, based on the results, the EORTC IN-PATSAT32 and PSCC-G can be recommended for use in cancer patients in the German-language region, while the German versions of the HCAHPS, NORPEQ, PPE-15 and PEACS can be recommended as generic PREMs.

## Supporting information

S1 Fig Quality of care model of the institute of medicine. (TIF)

S1 File Search algorithm. (DOCX)

S2 File Methods for the qualitative analysis of psychometric properties. (DOCX)

S1 Table Dimensions of patient centredness. (DOCX)

S2 Table PRISMA checklist. (DOCX)

S3 Table Study characteristics of all non-German Patient-Reported Experience Measures (PREMs) identified in the search. A) generic; B.) surgery-specific and C.) cancer care-specific PREMs.
(DOCX)

S4 Table Excluded full text articles. (DOCX)
